# Resveratrol Directly Binds to Mitochondrial Complex I and Increases Oxidative Stress in Brain Mitochondria of Aged Mice

**DOI:** 10.1371/journal.pone.0144290

**Published:** 2015-12-18

**Authors:** Naïg Gueguen, Valérie Desquiret-Dumas, Géraldine Leman, Stéphanie Chupin, Stéphanie Baron, Valérie Nivet-Antoine, Emilie Vessières, Audrey Ayer, Daniel Henrion, Guy Lenaers, Pascal Reynier, Vincent Procaccio

**Affiliations:** 1 Université d’Angers, Angers, F-49000, France; 2 Département de Biochimie et Génétique, CHU d’Angers, Angers, F-49000, France; 3 UMR CNRS 6214-INSERM U1083, Angers, F-49000, France; 4 EA 4466, Université Paris Descartes, Faculté de Pharmacie, Paris, F-75270, France; University of Alabama at Birmingham, UNITED STATES

## Abstract

Resveratrol is often described as a promising therapeutic molecule for numerous diseases, especially in metabolic and neurodegenerative disorders. While the mechanism of action is still debated, an increasing literature reports that resveratrol regulates the mitochondrial respiratory chain function. In a recent study we have identified mitochondrial complex I as a direct target of this molecule. Nevertheless, the mechanisms and consequences of such an interaction still require further investigation. In this study, we identified *in silico* by docking study a binding site for resveratrol at the nucleotide pocket of complex I. *In vitro*, using solubilized complex I, we demonstrated a competition between NAD+ and resveratrol. At low doses (<5μM), resveratrol stimulated complex I activity, whereas at high dose (50 μM) it rather decreased it. *In vivo*, in brain mitochondria from resveratrol treated young mice, we showed that complex I activity was increased, whereas the respiration rate was not improved. Moreover, in old mice with low antioxidant defenses, we demonstrated that complex I activation by resveratrol led to oxidative stress. These results bring new insights into the mechanism of action of resveratrol on mitochondria and highlight the importance of the balance between pro- and antioxidant effects of resveratrol depending on its dose and age. These parameters should be taken into account when clinical trials using resveratrol or analogues have to be designed.

## Introduction

Resveratrol (RSV), a redox-active polyphenol found in various plants, has a wide range of biological properties, including anticancer, anti-inflammatory, cardio- and neuro-protective effects. Its potential benefit on health has been mainly attributed to its antioxidant properties [1–3] and its interaction with the sirtuins, a class of histone deacetylase enzymes [4].

Mitochondria are key targets of RSV. Indeed, RSV modulates mitochondrial ROS production, mitochondrial biogenesis [5, 6], via its interaction with SIRT1 [7, 8], and energy metabolism via either transcriptional [9, 10] or enzymatic activation of SIRT3 [11, 12]. In addition, RSV also interacts with respiratory chain complexes, as the F0F1 ATP synthase [13], resulting either in an activation of the enzyme at low concentrations (i.e. picomolar to nanomolar) [14] or in an inhibition of its activity at higher doses (i.e. micromolar) [14, 15]. Zini et al. further showed that RSV also inhibited complex III [16]. Complex I (CI) plays a crucial role in maintaining mitochondrial homeostasis, not only through its role in the energy metabolism and the reactive oxygen species (ROS) production [17], but also by regulating the NAD+/NADH ratio [18]. Recently, we identified CI as a direct target of RSV, resulting in an increase of its enzymatic activity at low doses (i.e. 1–5 μM) [19]. Nevertheless, the mechanism of interaction between CI and RSV and its consequences on CI function remain to be determined. The recent crystallization of CI structure shed light on the subunit organization and the functional sites involved in its catalytic activities [20], enabling the *in silico* binding prediction studies. At a biochemical point of view, CI comprises three structural modules: N, Q and P [21]. The N-module is responsible for the oxidation of NADH into NAD^+^, generating 2 electrons and reducing the flavin mononucleotide (FMN). This latter is non-covalently bound to the NDUFV1 subunit (responsible for NADH oxidation and FMN binding). Then, the Q-module encompasses 7 iron-sulphur clusters that ensure the electron transfer and the final quinone reduction. Finally the P-module pumps protons across the inner mitochondrial membrane, with the energy released by the electron transfer [21]. In this study, we used a docking prediction to demonstrate the interaction between RSV and CI. We further used *in vitro and in vivo* analyses to demonstrate a direct binding of RSV at the CI nucleotide binding site of the NADH dehydrogenase module. We showed that RSV binding competed with the NAD+ fixation within the nucleotide binding pocket, increasing NADH oxidation at low doses (up to 5 μM RSV) but inhibiting CI activity at higher doses (50 μM RSV). We also demonstrated *in vivo*, in brain mitochondria of young and old mice, that RSV increased CI but led to oxidative stress in aged animals with low antioxidant defenses. Altogether, our results indicate that RSV interaction with CI affects differentially the mitochondrial metabolism depending on the dose and the cellular redox status.

## Materials and Methods

### Materials

Resveratrol (RSV), potassium hexacyanoferrate(III) (FeCN), and hexaammineruthenium (III) chloride (HAR) were purchased from Sigma-Aldrich (Lyon, France). NAD+ and NADH (grade II) were from Roche Applied Sciences (Lyon, France). Stock solutions of RSV were reconstituted at 50 mM in DMSO and stored at -20°C and protected from light. RSV quality was checked before all experiment by analyzing the absorption spectrum by UV/Vis spectroscopy in KH_2_PO_4_ solution at pH 7.2. RSV spectrum shows a single maximum absorption band between 304 and 310 nm [22].

### Docking simulation and NDUFV1 sequence analysis

Docking calculations were carried out using DockingServer [23]. The MMFF94 force field was used for energy minimization of RSV (ligand molecule) using DockingServer. Gasteiger partial charges were added to the ligand atoms. Docking calculations were carried out on Thermus thermophilus model of the N module of CI (subunits Nqo1, Nqo2, Nqo3) in both oxidized and reduced crystal structures (PBD ID: 4HEA for oxidized structure and 3IAM for reduced one respectively). Essential hydrogen atoms, Kollman united atom type charges and solvation parameters were added with the aid of AutoDock tools [23]. Affinity (grid) maps of 25×25×50 Å grid points and 0.375 Å spacing were generated using the Autogrid program [23]. AutoDock parameter set- and distance-dependent dielectric functions were used in the calculation of the van der Waals and the electrostatic terms, respectively. Docking simulations were performed using the Lamarckian genetic algorithm (LGA) and the Solis & Wets local search method. Initial position, orientation and torsions of the ligand molecules were set randomly. Each docking experiment was derived from 100 different runs that were set to terminate after a maximum of 2.500.000 energy evaluations. The population size was set to 150. During the search, a translational step of 0.2 Å and quaternion and torsion steps of 5 were applied. NDUFV1 amino acids conservation was performed with the NCBI multiple protein alignment tool Cobalt for four species (from *Homo sapiens* to the bacteria *Thermus thermophilus*) using standard parameters. Black highlighted residues represent amino acids which are conserved in all the species tested.

### Cell culture

This work was approved by the Ethical Committee of the University Hospital of Angers (CB 2014/02), and informed written consent was obtained from all participants. The two patients (P1 and P2) presented a Leigh syndrome related to a CI deficiency due to compound heterozygous mutations in the *NDUFV1 gene*: P1: c.156-2A>G, c.731A>G (p.Asn244Ser) and P2: c.1162+4A>C, c.1156C>T (p.Arg386Cys). Fibroblasts from patients and 5 healthy controls were cultured in 2/3 Dulbecco’s modified Eagle medium (DMEM-F12, PAN biotech, Germany), 1/3 Amniomax (Gibco, Invitrogen, Paisley, UK), supplemented with 10% fetal bovine serum (PAN biotech, Germany) at 37°C, 5% CO2. All experiments were conducted on fibroblast cultures between passages 6 and 25.

### Animals

The investigation was performed in agreement with the guidelines from Directive 2010/63/EU of the European Parliament on the protection of animals used for scientific purposes (authorization of the laboratory 00577) and the Guide for the Care and Use of Laboratory Animals published by the US National Institutes of Health (NIH Publication No. 85–23, revised 1996). The protocol was approved by the Institutional Animal Care and Use Committee (IACUC) and the Ethical committee of University Paris-Descartes (CEEA34.SB.008.12).

Male C57BL/6J strain mice (Janvier, Le Genest-St-Isle, France) were housed in a room at 24±2°C with a 12h/12h light-dark cycle, and diet and water *ad libitum*. Two groups were used: young (Y) (6 months), and old mice (O) (22 months). Twelve weeks before euthanasia, half of each group received a standard diet (M20 croquettes, (SDS®, Y Ctl and O Ctl groups), while the other half received a diet of M20 croquettes supplemented with 0.04% RSV (Yvery®, Y RSV and O RSV groups). The mice ingested 40 mg of RSV per kg and per day on average [24]. After 12 weeks of RSV diet, mice were anesthetized with isoflurane, before decapitation and dissection of the cerebral cortex. RSV accumulation in brain was checked according to Menet et al. [25]; the concentration of RSV and its derivates was 1.42±0.10 and 8.72±3.47 μmol/kg in Y and O RSV mice, respectively and undetectable in control groups.

### Mitochondria isolation and complex I solubilization

Mitochondria from mouse brain were isolated as described [26]. The entire protocol was performed at 4°C and completed in less than an hour. Isolated mitochondria from bovine heart were obtained from Mitosciences (Abcam, Paris, France). Solubilized complexes were prepared using n-dodecyl-β-D-maltoside [27].

### Mitochondrial enzymatic activities

The activities of the mitochondrial OXPHOS complexes were measured at 37°C with a UVmc2 spectrophotometer (SAFAS, Monaco). Activity of the NADH ubiquinone reductase (CI), succinate ubiquinone reductase (CII), ubiquinol cytochrome C reductase (CIII), cytochrome C oxidase (CIV) and citrate synthase (CS) were measured according to standard methods [28, 29]. NADH ubiquinone reductase (NUR) activity was assayed in KH2PO4 buffer (50mM, pH 7.5), containing 3.75 mg/ml fatty acid-free BSA and 0.1mM decylubiquinone. 0.1 mM NADH was added to initiate the reaction. Parallel measurements in presence of Rotenone (5μM) were used to determine the background rate. NADH:FeCN and NADH:HAR reductase activities were assayed in 20 mM KH2PO4 buffer (pH 7.5), with substrate and acceptor concentrations as previously described [30]. Background rates (without CI) were controlled for each experiment and RSV concentration. NADH:FeCN, NADH:HAR and NADH:Decylubiquinone oxidoreductions were monitored at 340 nm (ε = 6.22mM–1.cm–1).

### Mitochondrial respiration rates

Oxygen consumption was measured in Respiratory Buffer RB (225 mM sucrose, 10 mM Tris-HCl, 5 mM KH2PO4, 10 mM KCl, 1 mM EDTA, 4 mM MgCl2, 1 mg/ml fatty acid-free BSA, pH7.4) at 37°C using a high-resolution oxygraph (Oroboros, Innsbruck, Austria) on isolated brain mitochondria, (0.05 mg/ml) as described elsewhere [26, 31], using substrates of complexes I, I+III, II, IV and the glyceraldehyde-3-phosphate dehydrogenase (GAPDH) shuttle: CI substrates: 5 mM malate and 2.5 mM pyruvate; CI and II substrates: 5 mM malate, 2.5 mM pyruvate and 5 mM succinate; CII substrate: 10 mM succinate supplemented with 10 μM rotenone; CIV substrate: 5 mM N,N,N′,N′-tetramethyl-p-phenylenediamine reduced with 50 mM ascorbate. 20 mM glycerol-3-phosphate (G3P) was used to activate GAPDH shuttle. The active state of respiration (coupled-respiration) was initiated by the addition of a saturating ADP concentration (0.5 mM).

The direct impact of RSV or oxidative stress on mitochondrial functionality was analyzed on isolated brain mitochondria incubated during 30 minutes (1 mg in 0.2 ml buffer RB) with either DMSO (vehicle, final concentration 1/2000), H2O2 (10 nM, exogenous stress) or succinate without rotenone (10 mM, endogenous stress linked to reverse electron flow from CII to CI [32]).

### Western Blotting

SDS-PAGE electrophoresis and transfer to PVDF membrane were performed as described [33]. Rabbit anti-SOD2 (Mitochondrial matrix MnSOD, Abcam, Paris, France), mouse anti-N(epsilon)-(hexanoyl)lysine (HEL, Cosmobio, Tokyo, Japan) and anti—4 Hydroxynonenal antibody (4-HNE, Abcam, Paris, France) monoclonal antibodies were used for antioxidant enzyme and oxidation product detection, respectively. Detection of fluorescent secondary antibodies (Rabbit anti-mouse 680 nm and Goat anti-rabbit 790 nm) was performed using a LiCor Odyssey apparatus (Licor Biotechnology, Bad Homburg, Germany) with dual wavelength fluorescence detection (700 and 800 nm).

### Protein-SH content

Protein-SH content was measured on proteins from post-nuclear supernatant (50μg) using 5,5'-dithiobis(2-nitrobenzoic)acid (DTNB) as described [34], and the amount of protein-SH was calculated using reduced glutathione as standard (ε412nm = 13.6 mM^-1.^cm^-1^).

### Statistical analysis

Statistical comparisons between control and RSV mice were made with the Mann–Whitney *U* test. The Wilcoxon test was used for the analysis of paired data. Differences were considered statistically significant at *p*<0.05.

## Results and Discussion

### RSV is predicted to bind to complex I at the nucleotide binding site

**Fig 1:**
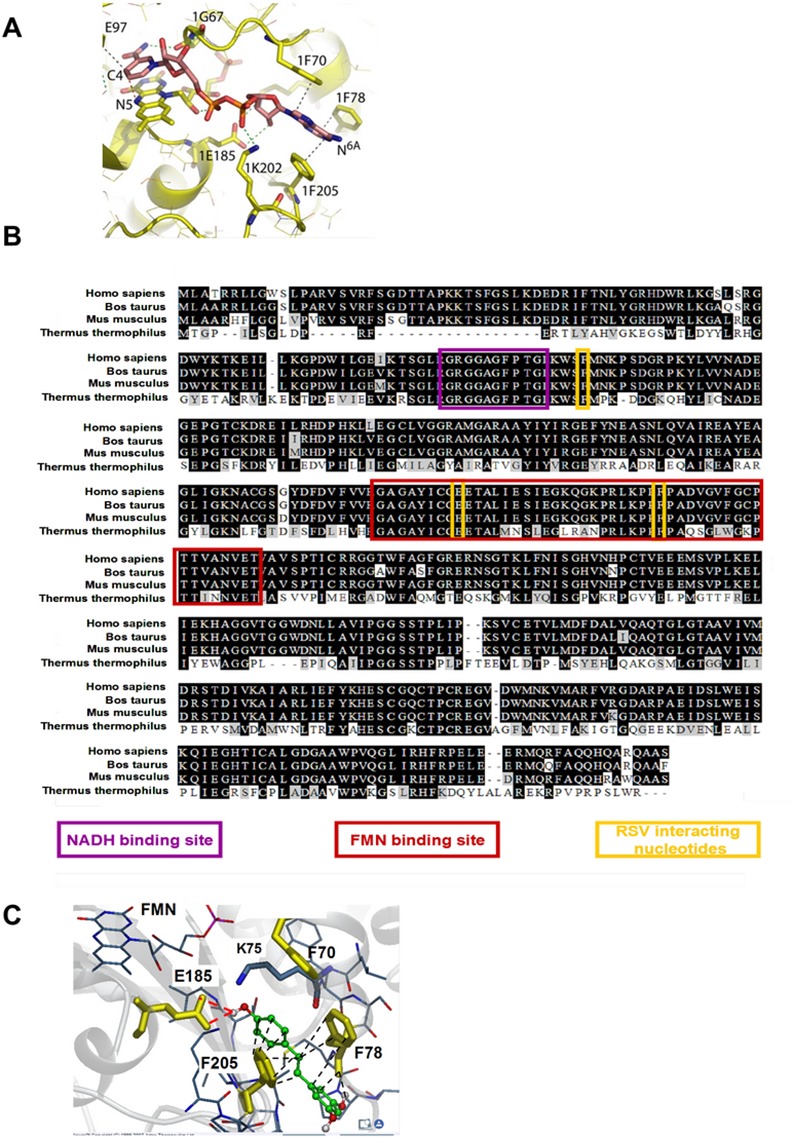
RSV binds to complex I at the nucleotide binding site. **(A) NADH binding site of complex I,** according to Sazanov et al. [37]. The bound NADH molecule is colored in pink. The FMN and amino acids that interact with the bound nucleotide are colored in yellow. The hydrophobic stacking interactions between the three phenylalanines and the adenosine moiety are represented by dashed gray lines. **(B) Boxshade alignement of the amino acid sequence of NDUFV1 (human nomenclature) from Homo sapiens, Bos taurus, Mus musculus and Thermus thermophilus** showing the conservation of the NADH and FMN binding sites. The conserved amino acids are highlighted in dark, the NADH binding site is boxed in purple, the FMN binding site in red, and the amino acids interacting with RSV are boxed in yellow. **(C) Occlusion of the flavin site through the binding of NAD(H) or RSV**. RSV bound to active FMN site in complex I from *Thermus thermophilus* (Protein Data Bank ID: 3IAM). The FMN and bound nucleotide are shown as sticks. RSV is colored in green. Amino acids that interact with the bound nucleotide are colored in yellow, with hydrogen bonding interactions shown by dashed red lines and hydrophobic stacking interactions by dashed dark lines.

To get insight into the effect of RSV on CI, a molecular docking study was used. While the structure of *Bos taurus* CI from heart mitochondria has been determined by single-particle electron cryo-microscopy [35], its X-ray high resolution structure is not yet available. Nevertheless, CI key subunits harboring the bio-energetic core functions are conserved from archae-bacteria to human [35, 36]. This is particularly the case for the 51 kDa, 24 kDa, 49 kDa, PSST and TYKY subunits (*B*. *taurus* orthologues of the human NDUFV1, NDUFV2, NDUFS2, NDUFS7 and NDUFS8 subunits, respectively) and the small domain of the 75 kDa subunit (orthologue of the human NDUFS1 protein) [32, 35, 37]. In this structure, the NADH binding site involves the aromatic rings of three conserved phenylalanines, i.e. Phe 70, 78, and 205 (Fig 1A) that stabilized the adenine ring of NADH or NAD+ by stacking interactions, while the carboxyl group of the conserved Glu185 interacts with the ribose of the molecules [20]. The flavin mononucleotide (FMN) is held in place by a hydrogen bonding network and interacts mostly with residues 175 to 220 [20, 35, 37] These residues involved in the nucleotide binding site are highly conserved throughout evolution (Fig 1B, red box), thus, we used the high-resolution structure of the CI NADH dehydrogenase module (N module) of *T*. *thermophilus* (PBD ID: 3IAM) for the docking study with RSV (Fig 1C).

Docked poses of RSV revealed its interactions with the nucleotide binding site, which involve hydrophobic and aromatic binding to two of the conserved phenylalanines, i.e. Phe78 and Phe205, and hydrogen bonds with Glu185 (Fig 1C). Thus, the RSV interaction with CI would imply three of the four amino acids involved in the stabilization of the adenine ring and ribose of the NAD(H) molecule; two of them being also involved in FMN binding (Glu185 and Phe205, Fig 1B, yellow squares). By contrast, docking study did not evidence any interaction between RSV and the amino acids stacking the nicotinamide head group of NAD(H), i.e. Gly67 and Glu97 [20]. The stacking of the nicotinamide head group is responsible for the stabilization of NADH binding, while it does not interfere with NAD+ stabilization [38], suggesting that RSV should more easily compete with NAD+ binding than with that of NADH.

**Fig 2:**
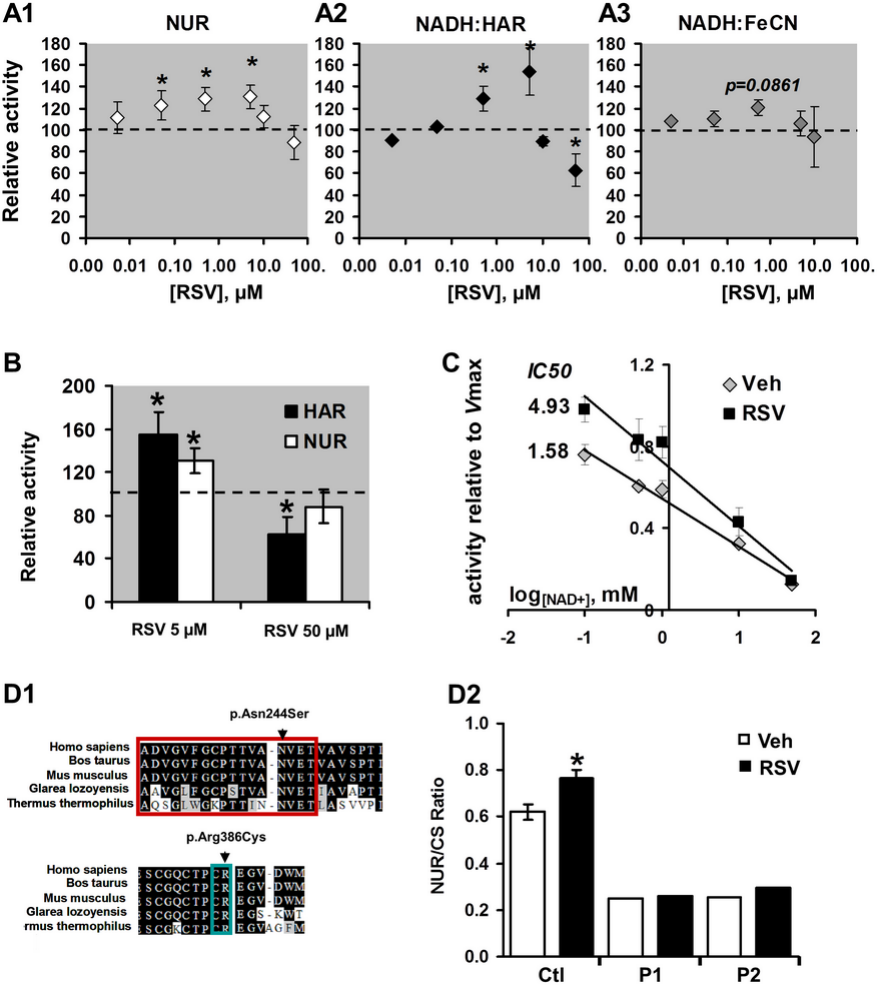
RSV binding to complex I modulates the kinetic of NADH oxidation. **(A) Effect of RSV on the NUR, HAR and FeCN enzymatic reactions catalyzed by complex I on isolated mitochondria. A1**: The rates of NADH:Ubiquinone oxido-reduction (the NUR reaction, A1), NADH:HAR oxido-reduction (the NADH:HAR reaction, A2) and NADH:FeCN oxido-reduction (the NADH:FeCN, A3) were measured on isolated CI in the presence of increasing RSV concentrations (5 nM to 50 μM). Reactions were started by the addition of NADH. Data are presented as a percentage of vehicle (DMSO), as mean ± *sem* of six independent experiments. The (*) indicates significant differences (p<0.05) compared with vehicle. **(B) Comparison of the effect of two RSV concentrations (5 and 50** μ**M RSV) on HAR and NUR reactions on isolated mitochondria**. Data are presented as a percentage of vehicle (DMSO) and as a mean ± *sem* of six independent experiments. The (*) indicates significant differences (p<0.05) compared with vehicle. **(C) Effect of RSV on the FeCN reaction and competition with NAD+.** The rate of the FeCN reaction was measured in the presence of either DMSO (vehicle) or 5 μM RSV and increasing NAD+ concentrations (0.1–50 mM). IC_50_ values for the inhibition of FeCN reaction by NAD+ were calculated from the curves normalized to maximal oxidation rates. Data are represented as mean ± *sem* of six independent experiments. The (*) indicates significant differences (p<0.05) compared with vehicle. **(D) Effect of RSV on complex I cells carrying NDUFV1 mutations in the nucleotide binding site.** D1: Position of the two exonic mutations on the NDUFV1 amino acid sequence. The NADH binding site is squared in red, and the Fe/S (N3cluster) interacting amino acids are squared in blue. D2: The NDUFV1 mutant fibroblast cells and five controls were incubated with 5 μM RSV, during 24h. The NUR activity was then measured on cell extracts. Data are expressed as the percentage of CI activity in vehicle treated cells and are represented as mean *± sem* of n = 5 control cells or as a mean of the triplicate analyses in mutant cells. The (*) indicates significant differences (p<0.05) between vehicle and RSV treated cells.

To challenge the docking results, solubilized CI preparations were used (Fig 2A, 2B and 2C) and incubated with 5 nM to 50 μM RSV, before assessing 3 different enzymatic reactions catalyzed by the different CI domains (Fig 2A): i) the decylubiquinone addition enables the measurement of the overall NADH Ubiquinone Reductase reaction (NUR, Fig 2A1), ii) the HAR (NADH:HAR reaction, Fig 2A2) and iii) the FeCN (NADH:FeCN reaction, Fig 2A3) addition enables the direct re-oxidation of the reduced FMN within the N module. At low doses (5 nM to 5 μM), RSV dose-dependently stimulated the NUR reaction, reaching a maximal effect at 5 μM (+35%, p<0.05, Fig 2A1). At higher concentrations, NUR activity progressively decreased to the vehicle value (RSV *vs* Veh: -15% for 50 μM RSV, Fig 2A1 and 2B). The HAR reduction was also stimulated by low RSV concentrations up to 5 μM (+48%, p<0.01, Fig 2A2), but was significantly inhibited at higher ones (-30% at 50 μM RSV, p<0.05, Fig 2A2). Finally, RSV did not significantly affect FeCN reduction (1 μM RSV: p = 0.09, Fig 2A3). For this latter test, the effect of 25 to 50 μM RSV doses could not be investigated due to a strong interaction between FeCN and RSV, leading to a direct NADH oxidation despite the absence of any enzyme (not shown).

The NUR reaction involves the NADH oxidation at the FMN site, followed by FMN re-oxidation by the FeS clusters and subsequent electron transfer to the quinone. By contrast, the HAR reduction does not involve FeS cluster chain or quinone reduction, and enables the measurement of the NADH dehydrogenase activity of CI [38]. Our results demonstrated that RSV effects on NUR activity can be reproduced and even amplified by the HAR addition (+ 58% for 5 μM RSV and– 36% for 50 μM RSV, Fig 2B) suggesting that the N-module is a molecular target of RSV, thus reinforcing the docking result. In addition, the effects of RSV were biphasic, first increasing up to 5 μM, then decreasing the turnover rate of the reaction at higher concentrations. The rate limiting step of the HAR reaction at low nucleotide concentrations is the HAR reduction, which one requires the presence of a negatively charged nucleotide bound to the reduced FMN [39]. RSV is known to easily lose its low-energy electrons, producing negatively charged species [40] among which phenoxide anion [41]. Thus, by increasing the occupancy of the reduced-FMN site and/or by increasing the local negative charge, low RSV concentrations may increase the rate of HAR reduction. However, at higher concentration, RSV may also compete with NADH at the nucleotide binding site, thus progressively inhibiting the rate of NADH oxidation, as seen at a concentration of 50 μM. By contrast, the FeCN reaction was not significantly modified by RSV, which could be explained by the fact that FeCN only reacts with a nucleotide-free reduced FMN [39].

Docking study suggested that RSV could compete more easily with NAD+ binding than NADH binding. To test this hypothesis, NADH:FeCN activity was measured in the presence of RSV and increasing concentrations of NAD+ (Fig 2C), a known weak competitive inhibitor of CI [38]. NAD+ addition alone inhibited the NADH:FeCN oxido-reduction, with an IC50 value of 1.58 mM (Fig 2C), while the presence of 5 μM RSV increased the IC50 value to 4.93 mM (Fig 2C), suggesting that RSV can displace NAD+ from its binding to the nucleotide binding pocket. Moreover, the higher was the NAD+ concentration, the lower RSV counteracted the inhibition of the NADH:FeCN reaction. Ultimately at 10 mM, the inhibition of the FeCN reaction by NAD+ was no longer modified by RSV, suggesting altogether a direct competition between RSV and NAD+.

The nucleotide binding pocket is located within the 51-kDa subunit corresponding to the human orthologue NDUFV1 [37]. To confirm the interaction between RSV and this site, we tested whether mutations of NDUFV1 modify the effects of RSV on CI. For this purpose, we used fibroblasts from two patients carrying compound heterozygous mutations in the NDUFV1 subunit P1: c.156-2A>G, c.731A>G (p.Asn244Ser) and P2: c.1162+4A>C, c.1156C>T (p.Arg386Cys). These mutations alter the FMN binding site in P1, or the FMN re-oxidation by altering the Fe/S cluster N3 in P2 (Fig 2D1). Both mutant cell lines presented a severe CI deficiency, with 30% residual NUR activity compared to control cells (Fig 2D2). The mutant cells and five control cell lines were treated with 5 μM RSV for 24h. RSV treatment resulted in a 30% increase of NUR activity in control cells, while no significant change was detected in NDUFV1-mutant cells compared to vehicle (+5% for P1 and +7% for P2, Fig 2D2). This result reinforces the hypothesis that the mechanism of action of RSV on CI activity involves an interaction with the FMN and nucleotide binding site.

### RSV targets specifically Complex I

**Fig 3:**
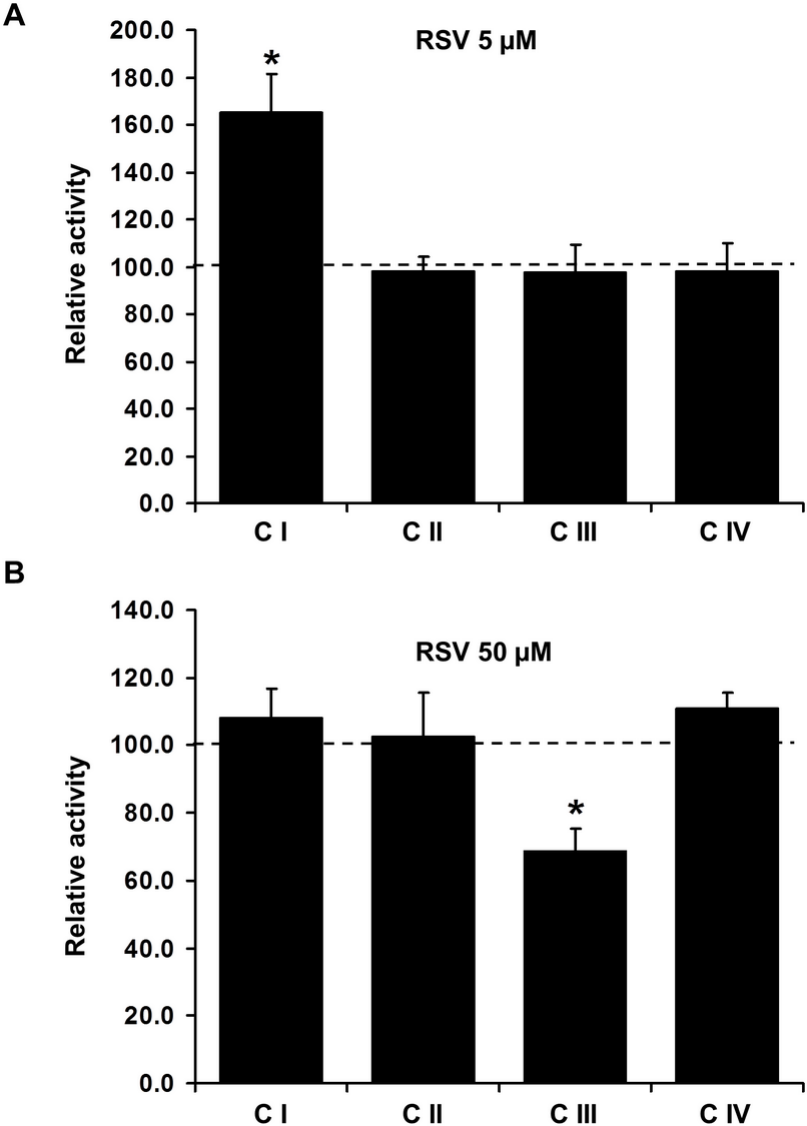
RSV specifically targets Complex I activity in brain mitochondria. **(A) Maximal activity of complexes in mitochondria incubated with 5** μ**M RSV. (B) Maximal activity of complexes in mitochondria incubated with 50** μ**M RSV.** Mouse brain mitochondria disrupted by frozen/thawed cycles were incubated with either vehicle (DMSO 1/2000) or RSV (50 μM) just prior analyzing complex I to IV activities. Data are expressed as the percentage of complex activities in vehicle treated mitochondria and are represented as mean *± sem* of n = 5 animals in duplicate. The (*) indicates significant differences (p<0.05) between vehicle and RSV treated mitochondria.

Then, we checked whether RSV also modifies the activity of other respiratory chain complexes (Fig 3). To perform these experiments, brain mitochondria were selected, as we found that RSV induces a +80% stimulation of NUR activity in this tissue, compared to +30% in heart mitochondria (S1 Fig), confirming previous observations of a high sensitivity of brain mitochondria to RSV [16, 42]. Frozen-thawed disrupted brain mitochondria were incubated with RSV prior to enzymatic measurements. As observed on solubilized CI, 5 μM of RSV significantly increased brain CI activity, while the activities of the CII, III and IV were not modified compared to vehicle (Fig 3A). At a higher dose (50 μM), the effect of RSV on CI activity was lost (Fig 3B) and the activity of CIII was significantly reduced (Fig 3B), the latter effect being related to a higher antimycin-insensitive rate and not to an overall decrease of the reaction rate (data not shown). This does not argue for a direct alteration of CIII catalytic properties, but rather indicates a non-specific interaction of RSV with CIII measurement, possibly by a direct reduction of oxidized cytochrome c or non-specific oxidation of the reduced decylubiquinone (DQH2). Previous studies also reported an inhibition of CIII by RSV [16, 42, 43]. For instance, Zini et al. suggested that RSV competes with DQH2 to bind CIII and directly accept electrons from this one [16]. In accordance with this hypothesis, Pshenichnyuk and Komolov have demonstrated that RSV can efficiently accept electrons [40] provided by leakage from CIII, forming semiquinone species. This mechanism would bypass the specific DQH2 re-oxidation and explain the apparent decrease of CIII activity.

Altogether, the results obtained *in silico* (Fig 1), *in vitro* on solubilized mitochondrial CI (Fig 2A) and on whole mitochondria (Fig 3), and *in situ* on treated cells (Fig 2D) point to a specific action of RSV on CI at low doses (up to 5 μM), through the interaction with the nucleotide binding site, increasing the NADH oxidation rate (NUR and HAR activities). However, at higher RSV concentration (50 μM), RSV lost the ability to induce CI activity and on the contrary tended to inhibit the NADH oxidation catalysed by the N module. This is in agreement with previous studies which showed an inhibition of the CI in mouse brain and liver mitochondria incubated with 25 μM RSV [16, 42]. Moreover, at high dose, RSV could also trigger some unspecific reactions with the respiratory chain, as revealed by CIII activity measurement, and could act as an electron carrier, bypassing CIII to provide electrons to cytochrome c. These results highlight the importance of the RSV doses when studying its effect on mitochondrial metabolism.

### RSV increases complex I activity in brain mitochondria of RSV fed mice but without improving respiratory chain function

**Fig 4:**
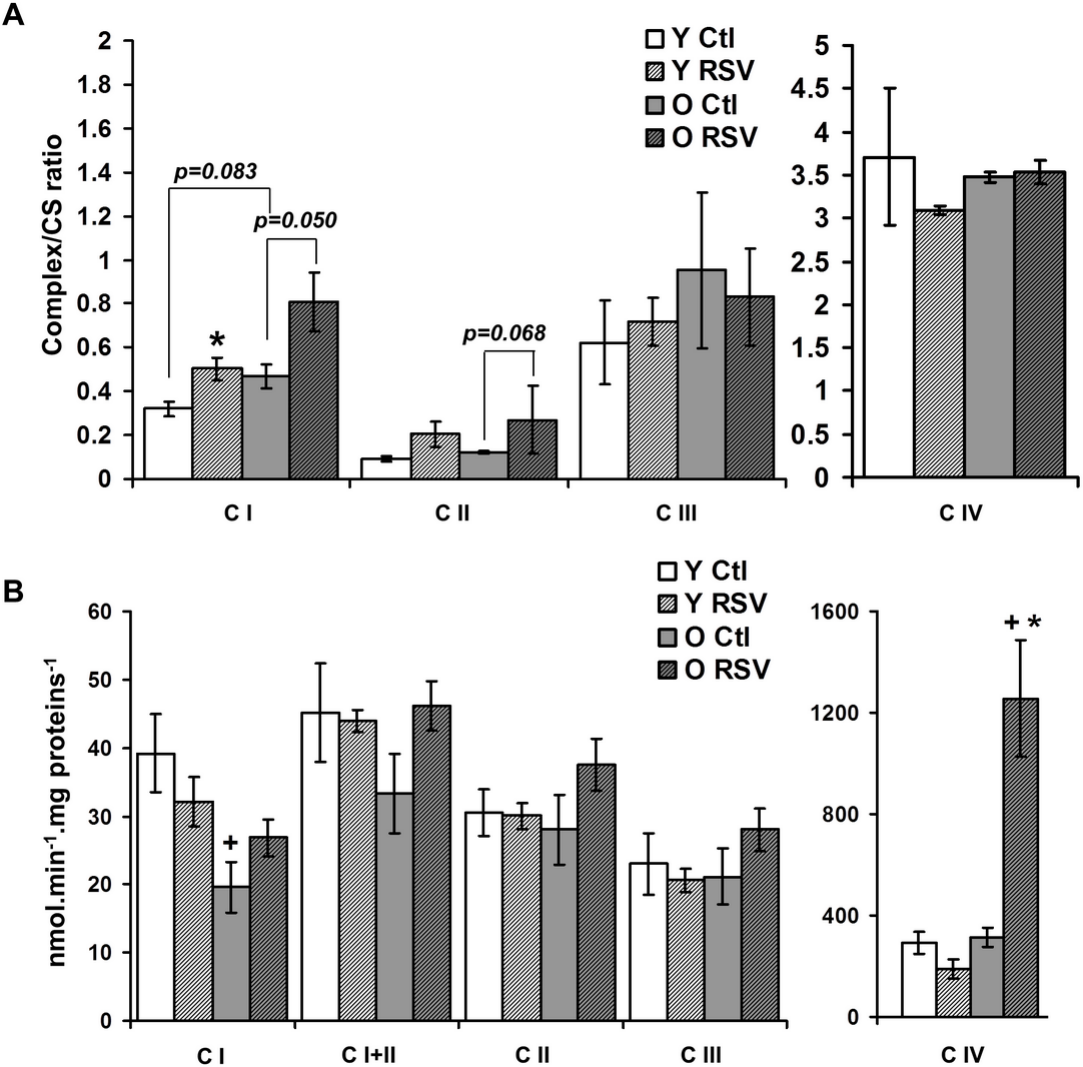
Functional consequences of RSV treatment on mitochondrial energy metabolism in brain mitochondria. **(A) Maximal enzymatic activities of respiratory chain complexes** were measured on isolated brain mitochondria from young (Y, white bars) and old (O, grey bars) control (Ctl, open bars) and RSV-treated (hatched bars) mice. Results are normalised to citrate synthase activity and represented as mean *± sem* of n = 5 animals in duplicate. The (*) indicates significant differences (p<0.05) between Ctl and RSV fed mice. **(B) Respiration rates measured on brain mitochondria** of young (Y) and old (O) Ctl (open bars) and RSV (hatched bars) treated-mice using different substrates of the respiratory chain and saturating ADP concentration (0.5 mM. Data are represented as mean *± sem* of n = 6 (young) or n = 5 (old) animals for each group. The (+) indicated a significant effect of aging (p<0.05).

Our data demonstrate that RSV targets CI activity *in vitro*. However, it remains to be determined if RSV could also stimulate or impair mitochondrial functions *in vivo*. For this purpose, the effects of RSV on respiratory chain function were studied on brain mitochondria from young (Y) and old (O) mice receiving a RSV-enriched diet for 12 weeks (Fig 4A). The use of a 0.04% RSV enriched diet (representing a daily intake of about 40mg/kg of RSV, [24]) was based on previous studies which have shown that similar RSV doses induced anti-aging effects [44], an improvement of mitochondrial oxidative metabolism under high fat diet conditions [6, 45] and antioxidant effects [46]. Moreover, we have shown in a previous article that a 12 weeks 0.04% RSV supplementation induced an increase in substrate supplies and respiratory chain function in liver of young and old mice [19]. Old animals were selected because of their higher susceptibility to oxidative stress, due to respiratory chain dysfunction and decreased antioxidant capacities [47]. In brain mitochondria from control O (O Ctl) mice, we did not observe any difference in respiratory complex maximal activities normalized to citrate synthase activity, compared to control Y (Y Ctl) mice (Fig 4A). However, the activities of complexes I, III and IV and citrate synthase were all reduced by roughly 25% in the O Ctl group compared to the Y Ctl group (data not shown). The reduced complex activities in O mice were in accordance with previous studies performed in brain mitochondria, showing that activities of complexes I and IV decrease linearly during aging [48–51]. RSV diet induced a significant increase in CI activity (p<0.05) in Y mice but only tended to increase CI in O mice (not significant, p = 0.05, Fig 4A), reinforcing our *in vitro* observations. RSV also tended to increase CII activity (Fig 4A, p = 0.0675) in Y mice, but not in O mice, a result that can be correlated with the stimulation of tricarboxilic acid cycle enzyme activities that was reported in liver from RSV fed mice [19]. Complexes III and IV activities were not significantly changed by the RSV diet.

**Fig 5:**
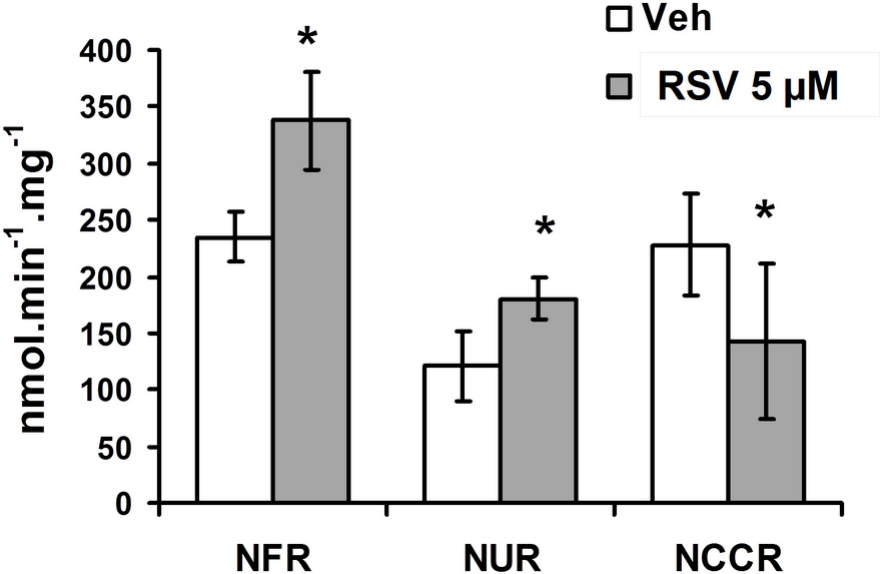
RSV increases the NFR and NUR activities but decreases the NCCR activity. Control young mice brain mitochondria were incubated with either DMSO (1/2000, vehicle) or 5 μM RSV (30 minutes) and NADH Ubiquinone Reductase (NUR), NADH:FeCN reductase (NFR), and NADH cytochrome C reductase (NCCR) activities were measured on incubated mitochondria. Data are represented as mean *± sem* of n = 3 animals in duplicate. The (*) indicates significant differences (p<0.05) between vehicle and RSV treated mitochondria.

We then determined if RSV effects on enzymatic activities *in vivo* were followed by functional change of the respiratory chain. Old mice showed a significant reduction of CI associated respiration rate compared with the Y ones (Fig 4B, O Ctl vs Y Ctl: - 47%, p<0.05). RSV diet increased maximal CI activity (Fig 3A), but did not change the respiration rates in both Y and O RSV fed mice (Fig 4B CI, 4B CII and 4B CIII). Nevertheless, in O-RSV mice, RSV diet strongly increased the CIV-linked respiration (Fig 4B +300% CIV, p<0.01), suggesting an increase in cytochrome c oxidase capacity. However, CIV maximal activity was unchanged in O-RSV animals (Fig 3A). Thus, these results suggest that the increased respiration could be due to an important electron leak in O-RSV mice. Indeed, under stress conditions, cytochrome c may also be involved in ROS detoxification, oxidizing O2-. to dioxygen [52]. The released electron can then be transferred to CIV, thereby stimulating the respiration rate [53]. In our study the high increase in CIV-supported respiration rate without any increase in its maximal activity could therefore result of such an antioxidant role of cytochrome c. Incubation of brain mitochondria with H2O2 or with succinate in absence of rotenone (a condition known to favor a reverse-electron flow to complex I generating ROS overproduction [32]), confirmed that oxidative stress led to increased CIV driven respiration (S2 Fig). The concept of an electron leak in O-RSV mice was further supported by the measurement of NADH cytochrome c reductase (NCCR, complexes I+III) activity, shown to be reduced when brain mitochondria were exposed to RSV, while the activities of NADH:FeCN reductase (NFR, NADH dehydrogenase activity) and NADH Ubiquinone Reductase (NUR) were significantly increased with 5 μM of RSV (Fig 5). Hence, the decreased NCCR activity suggested that RSV affects the electron transfer from CI to CIII, thus reinforcing the concept of an electron leakage at CI, which could trigger ROS overproduction.

A lower electron coupling has already been correlated to ROS overproduction in various experimental models [54, 55], in particular after RSV treatment [16, 43]. RSV interacts with the FMN/nucleotide binding site, considered as the major site of ROS production in CI [56–59] and increases the NADH dehydrogenase activity of the CI. The stimulation of NADH dehydrogenase activity by RSV could increase the reduced state of FMN and decrease the FMN/FMNH2 turnover, a condition favoring ROS production [16, 43]. Alternatively RSV could efficiently accept electrons, forming semiquinone species [40]. Thus, the interaction of RSV with the FMN/nucleotide binding site could initiate redox cycling reactions, as described for other electron acceptor compounds [16, 43], leading to ROS production.

### RSV induces anti-oxidant defenses in brain mitochondria

**Fig 6:**
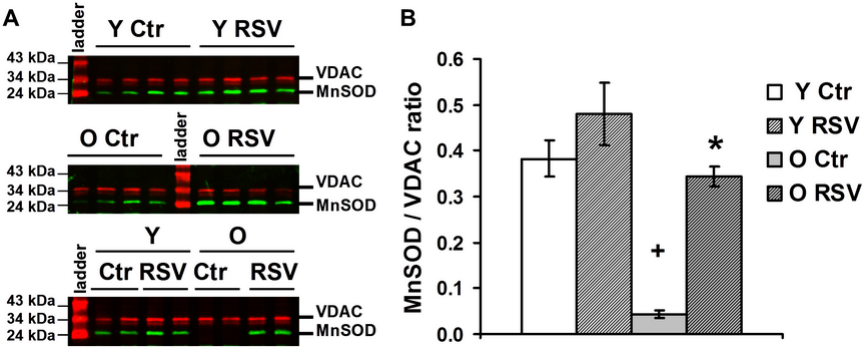
RSV increases the expression of anti-oxidant enzymes in brain mitochondria. Expression of MnSOD was analyzed by Western blotting (normalised to the quantity of VDAC protein) on mitochondrial fractions from young (Y) and old (O) control (Ctl) and RSV-treated mice. (A) Representative blot of two duplicate experiments on n = 4 animals. (B) Quantification of MnSOD expression. Data are represented as means ± *sem* of four animals in duplicate. The (+) indicates significant differences (p<0.05) between young and old animals and the (*) shows a significant effect of the RSV-diet (p<0.05).

To address the hypothesis of a higher ROS production within mitochondria from RSV fed mice, the mitochondrial manganese superoxide dismutase (MnSOD) expression was evaluated by Western blot in enriched mitochondrial fractions (Fig 6).

Our results revealed that aging reduces the protein expression of MnSOD (Fig 6), in agreement with previous studies describing a compromised antioxidant enzymatic status in brain mitochondria from aged rats compared to young animals [58]. The RSV diet increased MnSOD expressions in O animals (Fig 6). Such an induction of mitochondrial anti-oxidant defenses by RSV had already been described in other experimental models, exerting neuroprotective and anti-aging effects [60–63]. Recently, the so called hormetic effects of RSV were described, [64], showing that at low doses (<10 μM), RSV could act as an antioxidant, stimulating the cellular proliferation and the antioxidant response [5], while at higher concentrations (>50μM), it can become a prooxidant molecule, inducing cellular damages and decreasing cell viability [65–69]. In addition, it was reported that up to 5μM, RSV exerts a beneficial effect on sodium arsenite-induced cytotoxicity, promoting cell viability and proliferation, while it had opposite effects at a concentration of 20 μM [12]. These studies emphasized that low RSV concentrations reduce the level of oxidative stress, while higher concentrations are responsible for ROS overproduction shedding light on the importance of the redox cellular status and on the dose of RSV used. Moreover, the increased expression of anti-oxidant enzymes, as seen in the present study, could also reflect an adaptive response to the oxidative stress induced by the RSV itself.

### RSV increases anti-oxidant defenses but does not protect against oxidative stress in isolated brain mitochondria

**Fig 7:**
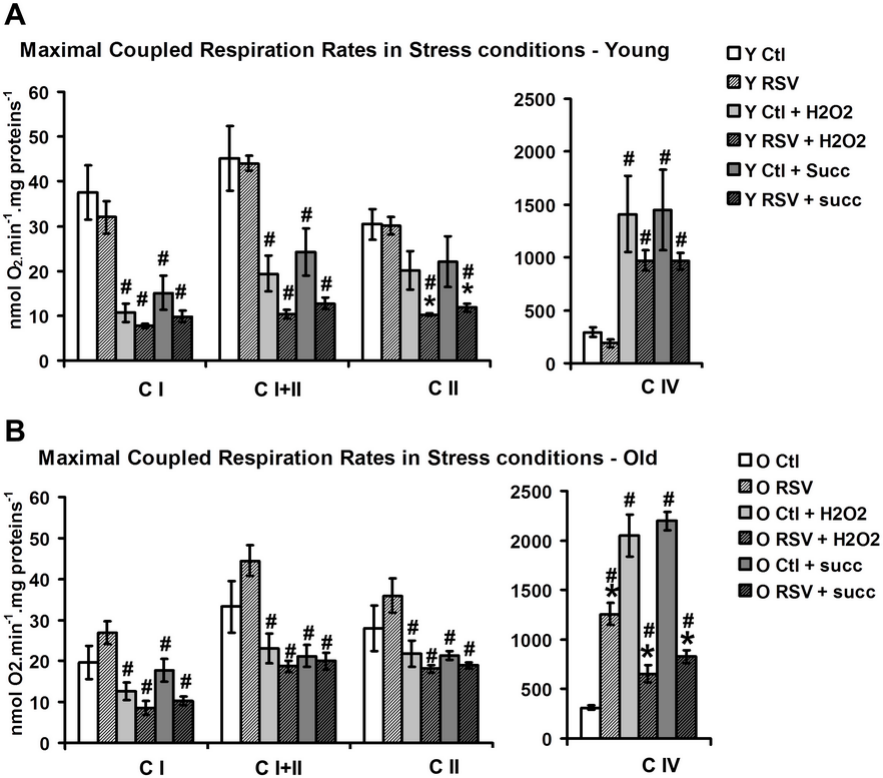
RSV does not prevent stress-induced inhibition of the respiratory chain. Brain mitochondria of young (A) and old (B) Ctl (open bars) and RSV treated (hatched bars) mice were incubated 30 minutes with 10 nM H_2_O_2_ (light grey) or 10 mM succinate (dark grey) without rotenone. Respiration rates were then measured using different substrates of the respiratory chain complex I to IV and saturating ADP concentration (0.5 mM). Data are represented as mean ± *sem* of five animals for each group. The (#) indicated a significant effect of H_2_O_2_ or succinate treatment, the (*) indicated a significant effect of the RSV diet (p<0.05).

To test the hypothesis of a protective effect of RSV against oxidative stress, isolated mitochondria were subjected to two different oxidative injuries, either using H2O2, to generate an exogenous oxidative stress, or using succinate without rotenone, to promote endogenous electron leakage from CI by a reverse electron flow [32] (Fig 7). In mitochondria from Y control mice, exposure to H2O2 induced a drastic inhibition of CI-linked respiration (-60%, Y Ctl + H2O2 vs Y Ctl) and a mild decrease in CII-linked respiration (-30%, Y Ctl + H2O2 vs Y Ctl). Similar effects were observed when incubating with succinate without rotenone (-56%, Y Ctl + Succ vs Y Ctl for CI and -30%, Y Ctl + H2O2 vs Y Ctl for CII, Fig 7A). Despite we did not directly measure the mitochondrial superoxide production in this experimental condition, the decrease of both CI and CII-linked respirations would argue in favor of the presence of an oxidative stress induced by reverse electron flow, as already reported [70]. In O mice, the respiration inhibition due to oxidative injuries was less pronounced (Fig 7B:- 30% for both CI and II respiration rates, Y Ctl + H2O2 and Y Ctl + Succ vs Y Ctl) possibly due to the lower basal respiration levels. The inhibition of mitochondrial respiration by either exogenous H2O2 or endogenous succinate without rotenone was not prevented in mitochondria from mice receiving a RSV-diet (Fig 7A and 7B, Y (O) Ctl + H2O2 vs Y (O) RSV + H2O2). The inhibition of CII-linked respiration due to the exposure of H2O2 or succinate was even stronger in Y mice receiving a RSV-diet compared to Y Ctl mice (-66% for Y Ctl RSV + H2O2 vs -30% for Y Ctl + H2O2, Fig 7A). Altogether, these results suggest that RSV does not protect against oxidative stress in isolated brain mitochondria and could even exacerbate the inhibition of respiration rates induced by an acute stress.

**Fig 8:**
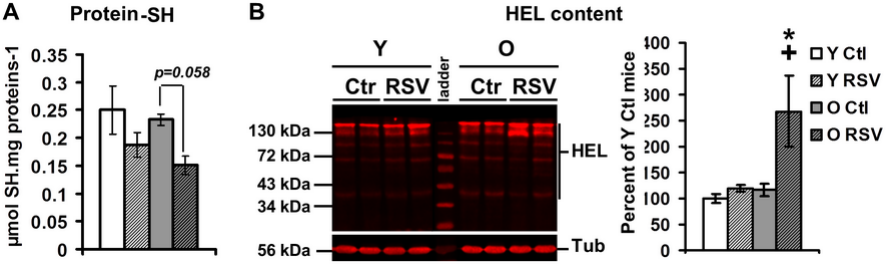
RSV increases oxidative stress markers. **(A) Protein thiols content** measured on brain homogenates of n = 4 animals for each group **(B) HEL-modified protein content on brain homogenates of young (Y) and old (O) control (Ctl) and RSV-treated mice**. Left, representative blot of two duplicate experiments on n = 2 animals; right, quantitation of HEL content by Western blotting on n = 6 animals. Data are represented as means ± *sem*. The (*) showed a significant effect of the RSV-diet (p<0.05).

We further addressed the hypothesis of the presence of a higher oxidative stress *in vivo*, in brain mitochondria of RSV-treated mice, by analyzing the level of oxidative cellular damages (Fig 8). Two markers of oxidative damages, the protein thiol (SH) content, a sensitive target of H2O2 oxidation [71], and the HEL and 4-HNE modifications, as biomarkers for the initial stage of peroxidation [72], were quantified by spectrophotometry and by Western blotting, respectively. While protein-SH content was slightly, not significantly, decreased in O RSV-treated mice (p = 0.058, Fig 8A), HEL-peroxided protein level was significantly increased in Old RSV-treated mice (Fig 8B, O Ctl vs O RSV p = 0.0104). Similarly, the content of 4-HNE modified proteins was increased in O RSV compared with the Ctl ones (p = 0.0104) (S3 Fig).

Taken together, these results strongly suggested that RSV diet induces a higher electron leak from the respiratory chain, leading to oxidative damages in the O mice, despite an increase in the expression of antioxidant enzymes such as MnSOD. Our results did not support the hypothesis of an hormetic action of RSV in our model as the increase in antioxidant defenses induced by RSV did not protect against but rather worsened the oxidative stress in aged mouse brain mitochondria. Because of high oxygen consumption, special membrane lipid composition and relatively low antioxidant defenses, brain tissues are highly sensitive to oxidative damages [73].

Here, we showed that in brain from O mice, RSV diet induced an electron leak from the respiratory chain, which overwhelmed antioxidant defenses in the context of aging with reduced antioxidant capacity.

Many studies indicated that RSV supplementation may convey resistance to oxidative stress [68, 74]. However, others studies show weak beneficial effects [75, 76] or even more detrimental side effects of RSV [77–79]. In particular, from a mitochondrial point of view, RSV has been shown to activate apoptosis, stimulating mitochondrial damages and ROS production [12, 80, 81]. It should be underlined that the mitochondrial toxicity could depend on the cell type, organism and/or the dose and duration of exposure to RSV [6, 82]. Most importantly, the effect of RSV could also differ on the presence or absence of cellular stress. Indeed, almost all experimental studies that pointed beneficial effects of RSV diets were conducted in a context of metabolic or/and oxidative stress (high fat diet, [6, 45, 83], diabetes or ischemia reperfusion [84]). However, these effects seem to vary depending on the stress context. Indeed, *in vivo*, the study by Rocha et al., [76] on RSV-treated rats (30 days, 1 mg/kg/day) demonstrated that RSV have beneficial effect in the animals with a high fat diet but rather displayed some potential adverse effects in the standard-fed diet (enhanced oxidized glutathione and decrease GSH/GSSG ratio). Similarly, in human, RSV supplementation does not improve metabolic function in non-obese women with normal glucose tolerance [85] and RSV was shown to blunt the positive effect of exercise training on cardiovascular biomarkers and maximal oxygen uptake on aged men [78].

Every antioxidant is in fact a redox agent and has the potential to become a prooxidant. Dietary polyphenols with phenol rings undergoing oxidation formed prooxidant radicals [86]. Resveratrol is known to form phenoxyl radical in the presence of metal cations [68]. In this context, RSV can react with of Cu(II) and induced DNA breakages. This prooxidant effect has also been observed with trivalent iron (Fe3+) in presence of H2O2 [86]. Aging is characterized by an accumulation of transition metal ions (such as iron [87] and copper [88]), a phenomenon that has been shown to be upregulated in neurodegenerative disorders [87]. In brain of old animals, the oxidative cellular context can therefore account for the major prooxidant effect of RSV. Thus assessing RSV effects on brain tissues represents a critical issue, as this drug has been envisioned as a neuroprotective molecule preventing neuronal damages in aging, Alzheimer’s and Parkinson’s diseases, and after cerebral ischemia [63, 89, 90].

## Conclusion

In summary, we showed that RSV binds to the CI nucleotide binding site, which either stimulates or inhibits its activity, according to RSV concentration. In addition, the targeting of CI by RSV stimulates key signaling pathways, including the antioxidant defenses. While the increase in ROS production would participate to the hormetic effects of RSV, in a context of reduced antioxidant capacity seen for instance in mouse aged brain, this process could also trigger damaging oxidative stress. Thus, this study emphasized that key parameters, such as the dose but also the age at the time of treatment, can modulate the intracellular and mitochondrial redox status, switching from beneficial to deleterious effects of RSV. Our study also shed light on the importance of a thorough characterization of the other possible molecular targets of RSV before moving further to clinical studies.

## Supporting Information

S1 Fig Comparison of RSV effect on complex I activity in brain and heart isolated mitochondria. Mice brain and heart mitochondria disrupted by frozen/thawed cycles were incubated with either vehicle (DMSO 1/2000) or in the presence of increasing RSV concentrations (5 nM to 5 μM) just prior analyzing the rate of NADH:Ubiquinone oxido-reduction (NUR reaction). Reactions were started by the addition of 0.1 mM NADH. Data are presented as a percentage of vehicle-treated mitochondria, as mean *± sem* of three independent experiments. The (*) indicates significant differences (p<0.05) compared with vehicle.
(TIF)

S2 Fig Measurement of TMPD-induced respiration rates in stress conditions. **(A)** TMPD-induced respiration rates (state IV, no ADP added) were measured in control brain mitochondria incubated during 30 minutes with either vehicle only (DMSO, 1/2000) or with10 nM H2O2, 10 mM succinate without rotenone (Succ) or 0.1 μM RSV (diluted in DMSO, 1/2000 final concentration). (B) The inhibition of TMPD-induced respiration by N-AcetylCysteine (NAC, 2 mM) added as an antioxidant was measured in control brain mitochondria incubated with vehicle (DMSO) or 0.1 μM RSV. Data are represented as mean *± sem* of five animals. The (*) indicated a significant effect of the treatment compared to vehicle condition (p<0.05).
(TIF)

S3 Fig RSV increases oxidative stress markers. **HNE-modified protein content on brain homogenate of young (Y) and old (O) control (Ctl) and RSV-treated mice**. Left, representative blot of two duplicate experiments on n = 2 animals; Right, quantitation of 4-HNE modified protein content by Western blotting on n = 6 young and n = 5 old animals. Data are represented as means ± *sem*. The (*) showed a significant effect of the RSV-diet and the (+) showed a significant difference between old and young mice (p<0.05).
(TIF)

## Abbreviations

Cyt c: Cytochrome c
FeCN: Potassium hexacyanoferrate(III)
HAR: Hexaammineruthenium(III) chloride
MnSOD: Manganese Superoxide Dismutase
NAD+: Nicotinamide Adenine Dinucleotide
NADH: Nicotinamide Adenine Dinucleotide Hydrate
NUR: NADH:(Decyl)Ubiquinone Reductase
ROS: Reactive Oxygen Species
RSV: Trans-resveratrol
