# Supplementary figures and images for: Resveratrol Directly Binds to Mitochondrial Complex I and Increases Oxidative Stress in Brain Mitochondria of Aged Mice
Source: PLoS One. 2015 Dec 18;10(12):e0144290. doi: 10.1371/journal.pone.0144290 (PMC4694087; doi:10.1371/journal.pone.0144290)

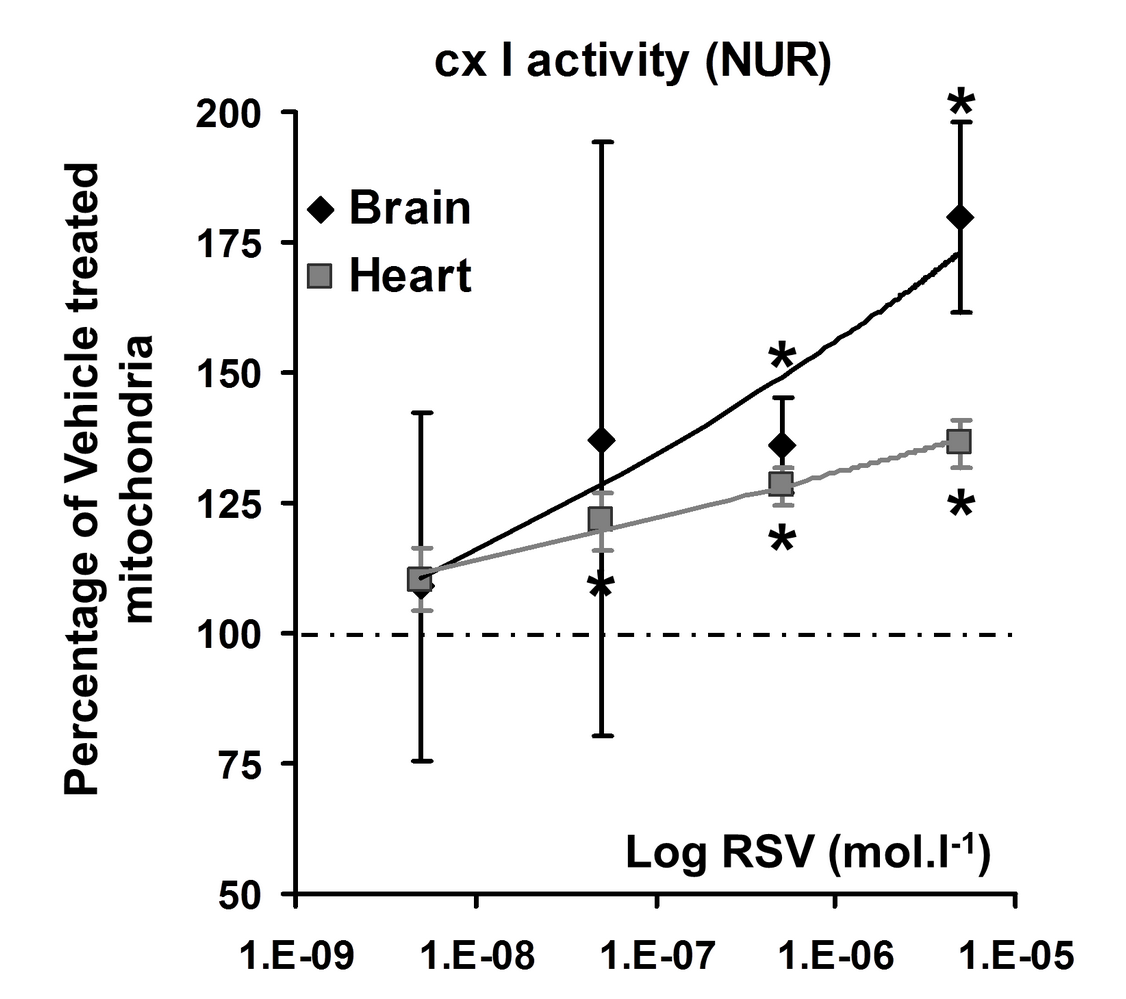

Supplement: S1 Fig — Mice brain and heart mitochondria disrupted by frozen/thawed cycles were incubated with either vehicle (DMSO 1/2000) or in the presence of increasing RSV concentrations (5 nM to 5 μM) just prior analyzing the rate of NADH:Ubiquinone oxido-reduction (NUR reaction). Reactions were started by the addition of 0.1 mM NADH. Data are presented as a percentage of vehicle-treated mitochondria, as mean ± sem of three independent experiments. The (*) indicates significant differences (p<0.05) compared with vehicle. (TIF) [file pone.0144290.s001.tif]

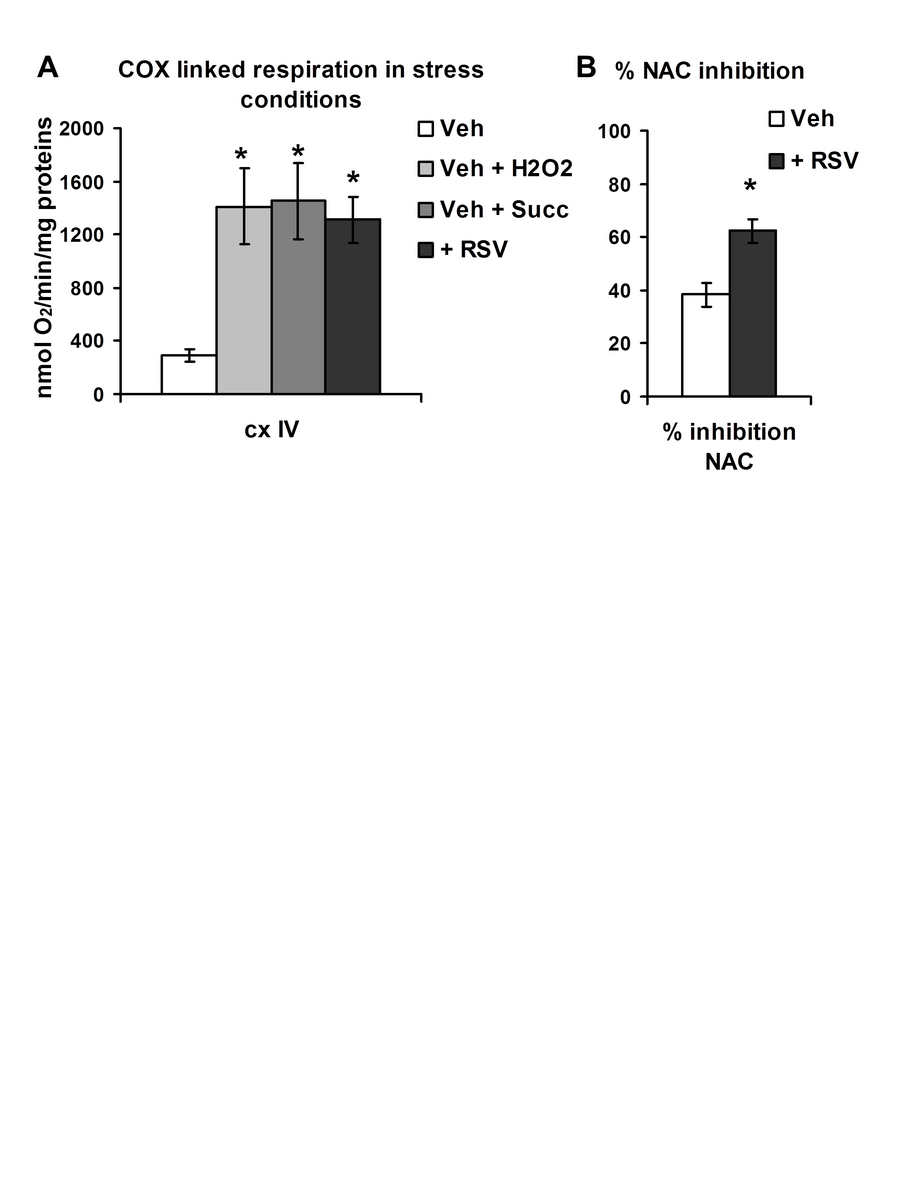

Supplement: S2 Fig — (A) TMPD-induced respiration rates (state IV, no ADP added) were measured in control brain mitochondria incubated during 30 minutes with either vehicle only (DMSO, 1/2000) or with10 nM H2O2, 10 mM succinate without rotenone (Succ) or 0.1 μM RSV (diluted in DMSO, 1/2000 final concentration). (B) The inhibition of TMPD-induced respiration by N-AcetylCysteine (NAC, 2 mM) added as an antioxidant was measured in control brain mitochondria incubated with vehicle (DMSO) or 0.1 μM RSV. Data are represented as mean ± sem of five animals. The (*) indicated a significant effect of the treatment compared to vehicle condition (p<0.05). (TIF) [file pone.0144290.s002.tif]

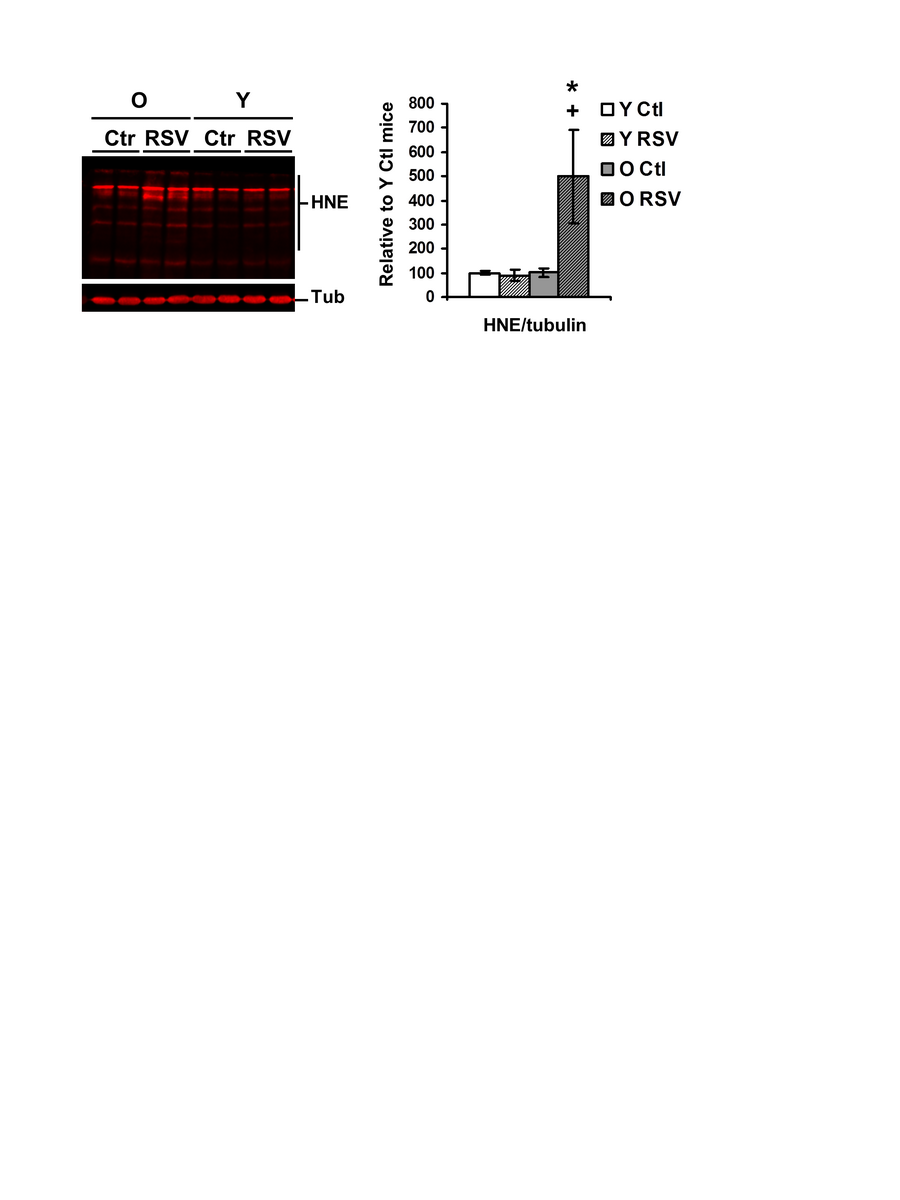

Supplement: S3 Fig — HNE-modified protein content on brain homogenate of young (Y) and old (O) control (Ctl) and RSV-treated mice. Left, representative blot of two duplicate experiments on n = 2 animals; Right, quantitation of 4-HNE modified protein content by Western blotting on n = 6 young and n = 5 old animals. Data are represented as means ± sem. The (*) showed a significant effect of the RSV-diet and the (+) showed a significant difference between old and young mice (p<0.05). (TIF) [file pone.0144290.s003.tif]
